# Validation of computerized diagnostic information in a clinical database from a national equine clinic network

**DOI:** 10.1186/1751-0147-51-50

**Published:** 2009-12-10

**Authors:** Johanna C Penell, Brenda N Bonnett, John Pringle, Agneta Egenvall

**Affiliations:** 1 Department of Clinical Sciences, Faculty of Veterinary Medicine and Animal Science, Swedish University of Agricultural Sciences, PO Box 7054, SE-750 07 Uppsala, Sweden; 2 Department of Population Medicine, Ontario Veterinary College, University of Guelph, Guelph, Ontario, N1G 2W1, Canada

## Abstract

**Background:**

Computerized diagnostic information offers potential for epidemiological research; however data accuracy must be addressed. The principal aim of this study was to evaluate the completeness and correctness of diagnostic information in a computerized equine clinical database compared to corresponding hand written veterinary clinical records, used as gold standard, and to assess factors related to correctness. Further, the aim was to investigate completeness (epidemiologic sensitivity), correctness (positive predictive value), specificity and prevalence for diagnoses for four body systems and correctness for affected limb information for four joint diseases.

**Methods:**

A random sample of 450 visits over the year 2002 (n_visits _= 49,591) was taken from 18 nation wide clinics headed under one company. Computerized information for the visits selected and copies of the corresponding veterinary clinical records were retrieved. Completeness and correctness were determined using semi-subjective criteria. Logistic regression was used to examine factors associated with correctness for diagnosis.

**Results:**

Three hundred and ninety six visits had veterinary clinical notes that were retrievable. The overall completeness and correctness were 91% and 92%, respectively; both values considered high. Descriptive analyses showed significantly higher degree of correctness for first visits compared to follow up visits and for cases with a diagnostic code recorded in the veterinary records compared to those with no code noted. The correctness was similar regardless of usage category (leisure/sport horse, racing trotter and racing thoroughbred) or gender.

For the four body systems selected (joints, skin and hooves, respiratory, skeletal) the completeness varied between 71% (respiration) and 91% (joints) and the correctness ranged from 87% (skin and hooves) to 96% (respiration), whereas the specificity was >95% for all systems. Logistic regression showed that correctness was associated with type of visit, whether an explicit diagnostic code was present in the veterinary clinical record, and body system. Correctness for information on affected limb was 95% and varied with joint.

**Conclusion:**

Based on the overall high level of correctness and completeness the database was considered useful for research purposes. For the body systems investigated the highest level of completeness and correctness was seen for joints and respiration, respectively.

## Background

Computerized information in medical databases offers potential for epidemiological and clinical research by providing, for example, longitudinal and cross-sectional data [1,2], finding cases[3,4] and determining incidence of disease [5]. However, whenever the primary purpose of collecting data differs from the specific goals of the research, the quality of the data must be addressed [6]. In human medicine, computerized diagnostic information has been evaluated for studies on specific diseases such as inflammatory bowel disease [7], venous thromboembolism [8], fractures [9] and autism [10]. Evaluation of computerized medical information has usually involved validating a database against either a patient survey or a paper record[11,12]. Moreover, validation of electronic patient records based solely on the contents of the clinical database has also been performed, for example by comparing morbidity data to recognised diagnostic standards to confirm diagnoses and identify further cases [13].

In veterinary medicine, computerized medical information has been used increasingly in equine research for retrospective studies of selected diseases [14-17]. However, evaluation of computerized veterinary medical records concerning completeness and correctness of the disease data is uncommon. One study evaluated the quality of data at a Canadian veterinary teaching hospital by comparing information in the computerized record to the paper medical files [18]. Further, the completeness of disease information in a dairy cow database based on veterinary reporting has been examined [19]. Evaluation of the agreement between computerized diagnostic information from canine and equine insurance databases and corresponding paper files/medical records has also been reported [20-22]. Unfortunately the insurance data used allowed only one diagnosis per registered insurance claim and captures only diseases for which the costs exceed the deductible. Thus there is a need for another source of clinical information for epidemiologic studies on horses that can also describe minor health issues (including prophylactic care) and register all co-morbidities.

The accuracy of a database containing disease information can be determined by assessing its completeness, defined as the proportion of problems in the veterinary clinical records (considering the clinical records as the gold standard) that were recorded in the database (i.e. epidemiologic sensitivity), and correctness, defined as the proportion of recorded disease events in the database that truly happened (i.e. positive predictive value). Both correctness and completeness are relevant when determining the utility of a database for research purposes.

An equine clinical database is maintained by a national network of equine clinics (ATG Equine Clinics Ltd, PO-161 89 Stockholm, Sweden, http://www.hastklinikerna.se). At the ATG (the Swedish Horse Racing Totalisator Board) equine clinics, horses of all types are examined and treated. The main aim of this study was to assess the completeness and correctness of the diagnostic information in computerized clinical records (CCR) in a sample of visits at these equine clinics by comparing the computerized information to the corresponding hand written veterinary clinical records (VCR). Further, factors related to correctness were investigated and, for four body systems, the accuracy (completeness and correctness), observed specificity, and prevalence were described. Finally, the correctness of information on affected limb for four selected joint problems was determined.

## Methods

### The clinics

In Sweden the company ATG (http://www.atg.se) was established by the government to ensure long-term financial stability for trotting and thoroughbred racing. The surplus generated by the company's business (e.g. managing betting activities), approximately 1.5 billion SEK/€150 million yearly, is returned to the horse industry. The ATG Equine Clinics were originally organized in the 1970's to offer qualified veterinary equine clinical service throughout the country. In 2002 the ATG Equine Clinics were owned by ATG and included 20 horse clinics throughout Sweden. They were day clinics (with no night or weekend services; predominantly outpatients) that accepted all types of horses with any problem that could be dealt with during office hours. A horse could come to a clinic for a health procedure (e.g. prophylactic care and health examinations) a disease event, or both. The company veterinarians (approximately 32 in 2002) have national (Swedish) certification in equine diseases or are training for the certificate. Thirteen of the clinics also conducted ambulatory visits, which accounted for 2.7% of the visits during the year of study. Satellite clinics (n = 2) with restricted open hours and staffed by the main clinic personnel were excluded from the sample frame.

### The clinical database and veterinary records

The data retrieved for the present study originates from a large Oracle database, which is operated by the head company ATG. The information in the database for trotters and thoroughbred racing horses and the equine clinics is owned jointly by three organizations: the Swedish Trotting Association, the Swedish Jockey Club and ATG Equine Clinics. The database includes information relating to racing horses in Sweden (e.g. demographic information on all racing horses in the country, racing related information such as trainer, racing starts, finished races, placing, race times, personal best, prize money won), and information on all visits (including the few ambulatory visits) to the ATG Equine Clinics. At each visit, the usage category of the horse was determined as racing trotter, racing thoroughbred or leisure/sport. Demographic information on the horse (name, sex, breed and birth date) was either retrieved from the racing horse information part of the large database (for racing horses) or entered into the database at the time of admission (for leisure/sport horses). Also, name, address and identification number of the owner (i.e. person responsible for payment) as well as information on the diagnoses and diagnostic procedures for each visit was recorded. The CCR for the visit further included a unique visit identification number, date of visit, at which clinic the horse was seen and the diagnostic and procedure information related to the visit. The information in the hand written VCRs required for processing the visit (i.e. the invoice, which includes diagnostic and procedure information) was transferred to the CCR by a veterinary assistant or receptionist with assistance by the veterinarian if deemed necessary.

### Sampling

Sampling was on the visit level. From all visits during 2002 (n = 51,987), visits with incomplete information on the payment-responsible person (name and/or full address missing) were removed (1,275 visits) as well as horses seen at the two excluded satellite clinics (another 1,121 visits) producing a sample frame of 49,591 visits (95.4% of all visits). To select the number of visits to include in the sample, sample size calculations were made using StatCalc and sample size for population surveys/descriptive studies in Epi Info (version 3.4.3.). The calculations were based on population size, estimated level of correctness (85%) and lowest acceptable correctness (80%) with a 95% level of confidence yielding a suggested sample size of 195 records. To adjust for non-retrieval and enable further investigations the sample size was increased to the maximum number of clinical records deemed by the former company head veterinarian as practical to retrieve (n = 450). To select the simple random sample of 450 visits, a computerized random-selection procedure was used (using command uniform, Stata. Stata9, Corp).

The CCRs of the selected visits were accessed. Copies of the corresponding VCRs were retrieved by mailed requests to the clinics. If the chosen visit was a follow up visit (as determined by the procedure information), a copy of the VCR for the first visit immediately prior to the randomly chosen visit (as determined by the procedure information for the visits regardless of the time period between the visits) was also requested. Reminder letters were sent one month after the initial requests. Further contact was made by telephone to retrieve maximal number of VCRs.

### Diagnostic and procedure information

At each visit, at least one diagnostic code corresponding to the disease problem of the horse was registered in the database. The clinics used an alpha-numerical hierarchical diagnostic coding system with each code consisting of a combination of letters and digits [23]. The first two letters assign system (e.g. joints, muscular, digestive) and localisation within system (e.g. fetlock, stifle), respectively, and the following one to four digits specify category (e.g. inflammatory, traumatic), sub category and specific diagnosis. Additional details on the diagnostic registry have been published previously [24]. When recording diagnostic information in the CCR the receptionist/assistant interpreted the written diagnostic information (in consultation with the veterinarian when necessary) and entered the corresponding diagnostic code into the CCR or, when available, transferred the specific diagnostic code specified by the veterinarian in the VCR to the CCR. All records (CCR and VCR) had at least one problem/diagnosis recorded per visit. For each diagnosis recorded, information on affected limb (right/left, front/hind, both/all) could also be registered when relevant (e.g. for joint inflammation). At least one procedure code was recorded per visit. The procedure codes assigned the procedures undertaken during each visit, (e.g. flexion test, sedation) and also determined if the visit was a first visit or a follow up visit to the clinic.

### Data handling

Visits lacking a VCR were excluded from further analyses (n = 54). The information sampled from the CCR was transferred into a database (MS Access, Microsoft Corporation, Redmond, WA 98052-6399, USA) and information from the available VCRs was entered manually by the first author, who also evaluated the records. The diagnostic information in the VCR was interpreted and transferred to the database blinded to the diagnostic information in the CCR. Data entries were checked repeatedly to ensure consistency in evaluation. The number of problems/diagnoses in the CCR and VCR for each visit (i.e. the chosen visit, not including first visit information if a follow up visit was selected) was determined by counting problems/diagnoses in each source. For VCRs, the complete clinical notes for the chosen visit were evaluated to determine the number of problems identified. Also, if a more specific diagnosis was present in the VCR, the related clinical signs were not counted as separate problems (e.g. fetlock joint inflammation and lameness in the same extremity was counted as one problem). Each specific diagnosis was only counted once per visit (even if different extremities had the same problem, e.g. carpal joint inflammation). The organ system(s) (e.g. joints) was determined both in the CCR and the VCR, either based on the exact code (i.e. the first letter in the diagnostic code) or, for the VCRs lacking a code, the written information. The system information was categorized into six categories based on the first letter of the diagnostic code recorded; joints, skin and hooves, respiratory, skeletal, whole body, and other (including the original systems cardiovascular, digestive, reproductive, ear and eye, muscular and neurological).

### Evaluation of correctness and completeness

The VCR was used as gold standard. Data in the CCR were evaluated relative to the recorded diagnoses in the VCR. For four selected joint diseases (fetlock, carpal, stifle and hock joint inflammation), information on affected limb from the CCR and the VCR was compared. For the selected visits with available VCR, correctness was calculated based on the number of problems/diagnoses recorded in the CCRs, whereas completeness was assessed based on the number of problems/diagnoses present in the VCRs. Correctness of diagnostic information was assessed using semi-subjective criteria (table 1) under the categories "total correctness", "partial correctness", and "absence of correctness". In the analyses, correctness included the categories total and partial correctness from the initial assessment. For each recorded diagnosis in the CCR, the level of correctness was assessed as: total correctness when the recorded diagnosis in the CCR corresponded well to the information in the VCR, partial correctness when the level of correctness was less satisfactory, or absence of correctness for incorrect information present in the CCR. In general, when the unspecific designation "without diagnosis" ('AA009' in system whole body) was recorded in the database, correctness was considered partial if there was either a specific or an unspecific/unknown diagnosis written in the veterinary notes. The completeness of the CCR (i.e. the ability of the CCR to capture all diagnostic information in the VCR) was assessed for each diagnosis/problem in the VCR (related clinical signs being excluded as problems if a more specific diagnosis was present, as described above) by determining if each diagnosis/problem in the VCR was also recorded in the CCR. The completeness was evaluated both in total and per clinic.

**Table 1:** Definition of criteria used to assess correctness

Correctness		Absence of correctness
	
Total	Partial	
The same diagnostic code was used (n = 294)	Slightly more precise information in the CCR (n = 12)	A diagnosis unrelated to the condition described by the VCR had been recorded in the CCR (n = 12)
The diagnostic code in the CCR corresponded to the diagnostic information in the VCR (n = 90)	"Without diagnosis" in the CCR and non-specific information in the VCR (n = 39)	Specific diagnosis in CCR despite non-specific diagnostic information in VCR (n = 26)
Diagnosis in the CCR was similar but less exact than the VCR (n = 1)	"Without diagnosis" in the CCR and specific information in the VCR (n = 17)	

Definition of criteria used for assessing correctness of diagnostic information in computerized clinical records (CCR) compared to the corresponding veterinary clinical records (VCR) and number of cases in each category in a sample of visits (in 396 visits including 491 individual diagnoses in the CCR) at 18 horse clinics in Sweden during 2002.

For four systems (joints, skin and hooves, respiratory and skeletal) the presence/absence of each system in every visit was determined for both sources, based on the first letter in the assigned diagnostic code(s), or for the VCR, the handwritten information. Analysis of correctness of information on affected limb was evaluated by assessing the proportion of observations with a certain limb affected according to the CCR that, according to the VCR, truly had a problem in that limb.

### Data analyses

Descriptive analyses were presented on the following variables: type of visit (first or follow up visit), gender (gelding, mare or stallion), usage category (racing thoroughbred, racing trotter or leisure/sport horse), whether a diagnostic code was explicitly written in the VCR, system (categorized as joints, skin and hooves, respiratory, skeletal, whole body and other), and on individual clinic basis. Information in the CCR on characteristics (i.e. the proportion that were first visits) of records with non-retrievable VCRs was compared to the information in records with available VCR.

All analyses were performed in Stata (Stata Special Edition, version 9.0, StataCorp, College Station, TX 77845, USA) with the exception of the model variation for the random effects logistic model, which was performed in MLwiN (version 2.0, Centre for multilevel modelling, Institute of Education, London WC1H 0AL, UK). The 95% confidence intervals (95% CIs) for correctness on diagnostic information were calculated using the two-tailed exact binomial test. The Pearson chi squared test was used to investigate the difference between the proportion of first visits for available and unavailable records based on information in the CCR and, for available records, whether the proportion of visits with one diagnosis registered varied between first and follow up visits. Further, the same test was used to investigate the difference in proportion of correctness on diagnostic information for first and follow up visits, for whether a diagnostic code was explicitly written in the VCR and for first and follow up visits for system joint, respectively.

Completeness (i.e. epidemiologic sensitivity; the proportion of those recorded as diseased in the VCR that were similarly recorded in the CCR, with 95% CI), correctness (i.e. positive predictive value; the proportion diseased according to CCR that were disease positive according to VCR), specificity (the proportion of disease negatives in the VCR that were similarly-recorded in the relevant CCR) and prevalence by system were calculated for the systems with >25 cases (>5% of all diagnoses recorded in the CCR) according to the CCR (excluding system whole body/unspecified).

The outcome correctness (yes/no) on diagnostic information (both total and partial correctness categorized as "yes") was modelled using logistic regression (using the logit and glamm commands in Stata). Due to clustering of diagnoses within visit in part of the data (18%) and with few observations within each cluster (2-5 diagnoses), one diagnosis was randomly selected to represent that visit in the final logistic model. Hence, the number of observations equalled the number of visits (n = 396). Explanatory fixed factors included whether an explicit diagnostic code was present in VCR, whether it was a first or a follow up visit, system diagnosis in CCR (categorized as joints, skin and hooves, respiration, skeletal and other), gender and usage category. All variables with p < 0.2 were eligible for inclusion in the multivariable model. Clustering of diagnoses within clinics was investigated and clinic was added as a random factor to the fixed effects model. Clustering of diagnoses within veterinarian was not investigated as this information was not accessed from the clinic database and was incomplete in the VCRs. The two-way interactions between dichotomous explanatory variables were considered for inclusion. Variables were also considered as confounders and, although not meeting the inclusion criteria of p < 0.20, were retained in the model if other estimates changed by >20% [25]. Model reduction was by manual backwards elimination of the variable with largest non-significant p-value, followed by re-running the model. The final p-value was set to 0.05. The significance of the random effect (i.e. clinic) was determined by comparing the models with and without inclusion of the random effect. This was done by a likelihood ratio test where the difference in log likelihood between the full and reduced model was multiplied by two and the value was compared to a χ^2^-distribution with 1 df [26]. The between clinic variation was estimated by dividing the random factor variance with (the random factor variance+π^2^/3), according to[27]. Model fit was addressed by the Hosmer-Lemeshow goodness-of-fit test with the data partitioned into 8 deciles, by assessing the predictive ability of the model evaluating the receiver operating characteristic curve (ROC curve), and by visual examination of diagnostic plots as outlined by Hosmer and Lemeshow (2000)[28]. Plots of Pearson residuals (*r*), leverage (*h*), delta beta (Δ*β*), delta deviance (Δ*D*) and delta chi2 (Δχ^2^) versus the predicted values were constructed and evaluated. The impact of outliers was assessed by running the model without the observations and comparing the coefficients between this model and the model using all observations.

## Results

### The sample

Of the 450 visits selected, copies of 396 hand-written VCRs were available, giving an overall retrieval rate of 88%. The number of copies of VCRs requested per clinic varied from 4 to 55. The median retrieval rate per clinic was 93% and varied from 25% (found one record of four) to 100%. Of the available records, one individual horse contributed two visits. The non-available records (n = 54) each had one diagnosis registered in the CCR database distributed as "without diagnosis" (n = 26), "prophylaxis" (n = 21), "healthy" (n = 3), lameness without further clinical signs, acute dermatitis, normal variation upper airways and acute bronchitis (all n = 1). Comparing unavailable and available records with respect to the information in the CCR showed that the proportion of first visits was significantly larger among the records with unavailable VCR than among the records with available VCR (98%, n = 54 versus 71%, n = 396, p < 0.001).

### Descriptive statistics

#### CCR

Leisure/sport horses were the most common horse type seen at the visits (n = 210), followed by racing trotters (n = 161) and racing thoroughbreds (n = 24). Two hundred and eighty two (71%) of the 396 visits were first visits and 114 were follow up visits. Table 1 shows the number of cases in each category in the assessment of correctness of diagnostic information for the diagnoses recorded in the CCR. For the 17 cases with "without diagnosis" recorded in the CCR, and where specific information was available in the VCR, 15 cases were related to health procedures such as teeth floating, castration and inspection for health certificate. In total there were 491 diagnoses registered in the CCR for the 396 visits with 323 (82%) visits having one diagnosis registered, 54 (14%) having two, 17 (4%) having three, 1 (0.3%) having 4 and 1 having 5 diagnoses registered. The median number of diagnoses per visit was 1. Of the 491 diagnoses registered, 338 (69%) were registered during first visits and 153 at the follow up visits. There was a significant difference (p < 0.005) in the proportion of visits with one diagnosis registered between first visits and follow up visits (86% and 71%, respectively). The designation "without diagnosis" was the single most frequently recorded diagnosis (16%), followed by fetlock inflammation (15%) and carpal joint inflammation (9%). The proportion of "without diagnosis" registered in the CCR varied between clinics, from 0 to 82% (median 15%).

#### VCR

In contrast, according to the VCR, there were 522 disease problems; 294 visits had one diagnosis recorded, 80 had two, 21 had three and 1 visit had 5 diagnoses recorded. Two hundred and thirty one (58%) records had a diagnostic code explicitly written out in the VCR. Of the 396 visits, 341 (86%) had the same number of problems noted in both sources. In 9 cases there were more problems noted in the CCR compared to the VCR whereas in 46 cases fewer problems were noted in the CCR compared to the VCR. For the 46 occasions where the CCR had one less problem recorded compared to the VCR, the most common problem not transferred to the CCR related to pointed enamel ridges (i.e. procedure teeth floating, n = 10).

Similar to the CCR, for the VCR the most recorded diagnosis overall was "without diagnosis", which was also the sole registered diagnosis for 75 visits and recorded together with another diagnosis for 2 visits. The procedures registered for "without diagnosis" (i.e. actions taken when this diagnosis was recorded) included, for example, lameness/locomotor evaluation (n = 16); radiographic examination (n = 14); blood analysis (n = 12); mouth check-up with/without teeth floating and/or extraction of wolf teeth (n = 12); selling of medicine and material (n = 11); castration (n = 5); endoscopic examination (n = 4) and bacteriological/virological analysis (n = 4). Sedation was recorded on 21 occasions and invariably together with another method such as radiographic examination or mouth cavity inspection/dental work.

### Evaluation of completeness and correctness; CCR versus VCR

Of the 522 disease problems noted in the VCR, 475 were also found in the CCR giving an overall completeness of 91% (475/522) with a between clinic range from 77% to 100%. The correctness on diagnostic information in the CCR was 92% (n = 453/491) and varied between clinics from 69% to 100%. If diagnostic information from the first visit (available for 28 visits of the 30 follow up visits included in the "absence of correctness group" above) was also included the overall correctness was 97%.

There was a significant difference (p < 0.005) in the degree of correctness for records with and without a specific diagnostic code written in the VCR (99% and 81% correctness, respectively) and for diagnoses recorded at first visits compared to follow up visits (97% and 80%, respectively). The correctness was similar for the three types of horses (91% for categories leisure/sport horse and racing trotter, 90% for racing thoroughbred) and also for the three genders (91% for geldings and mares, 90% for stallions).

Assessment of accuracy (completeness and correctness) and specificity between the CCR and the VCR and prevalence in each source for the four most common systems is shown in table 2. For joints, there was significantly higher correctness in first visits compared to follow up visits (98% versus 81%, p < 0.005). The distribution of correctness for affected limb information is shown in table 3. In total, information on affected limb was available for 129 diagnoses at 92 visits in both sources. The overall correctness on information on affected limb was 95% (124/129) and varied from 95% (fetlock and carpal inflammation) to 100% (stifle and hock joint inflammation).

**Table 2:** Evaluation of four body systems

		Joints	Skin and Hooves	Respiration	Skeleton
Number of records	System diagnosis present in both VCR and CCR	114	27	24	25
	System diagnosis in CCR, not in VCR	15	4	1	2
	System diagnosis in VCR, not in CCR	11	9	10	9
	No diagnosis for this system in either VCR or CCR	256	356	361	360
Tests	Completeness (%)^1^	91	75	71	74
	95% CI for Completeness^2^	85,96	58,88	53,85	56,87
	Specificity (%)^3^	95	99	98	99
	Correctness (%)^4^	88	87	96	93
	Prevalence in VCR (%)	32	9	9	9
	Prevalence in CCR (%)	33	8	6	7

Number of records for four body systems in the computerized clinical record (CCR) and the veterinary clinical record (VCR) and test results evaluating the ability of the CCR to accurately reflect the VCR (assuming the VCR is gold standard) in a sample of visits (n = 396) at 18 equine clinics in Sweden in 2002.

^1 ^defined as the proportion of problems in the veterinary clinical records (which were considered the gold standard) that were recorded in the database

^2 ^exact confidence interval

^3 ^95% CI varied between 91,97 to 98,100

^4 ^defined as the proportion of recorded disease events in the database that truly occurred according to the veterinary clinical record (i.e. the gold standard)

**Table 3:** Distribution and correctness of information on affected limb for four joint diseases

	Fetlock			Carpal			Stifle			Hock		
Limbs	CCR (same limb^1^)	VCR	Correct^1 ^%	CCR (same limb^1^)	VCR	Correct^1 ^%	CCR (same limb^1^)	VCR	Correct^1 ^%	CCR (same limb^1^)	VCR	Correct^1 ^%
Left fore only	12(12)	12	100	23(22)	23	96	-	-	-	-	-	-
Right fore only	10^2^(9)	11	90	9(8)	8	89	-	-	-	-	-	-
Both fore	30(28)	29	93	10(10)	11	100	^-^	^-^	-	-	-	-
Left hind only	5(5)	5	100	-^3^	-	-	3(3)	3	100	2(2)	2	100
Right hind only	4(4)	4	100	-	-	-	5(5)	5	100	6(6)	6	100
Both hind	1(1)	1	100	-	-	-	4(4)	4	100	4(4)	4	100
All four	1(1)	1	100	-	-	-	-	-		-	-	-
Total	63	63	95	42	42	95	12	12	100	12	12	100

Distribution of information on affected limb in the CCR and the VCR, the number of same affected limb recorded in both sources and the correctness of the affected limb information in the CCR (with the VCR as gold standard) (evaluated for each specific case within diagnosis and then summarized within joint) for four joint diseases (n = 129) in a sample of visits at 18 horse clinics in Sweden during 2002.

^1 ^Number of observations that had same information on affected limb noted in both sources

^2 ^Evaluated for each specific case and then summarized within joint disease, for example of the 10 cases of fetlock inflammation in the CCR that had right fore limb only noted as affected limb, 9 had the same affected limb noted in both sources; correctness for affected limb information in the CCR (i.e. fetlock joint inflammation in right fore limb) was 90%.

^3-^No observations

### Logistic regression analysis

In the univariable analysis three variables passed the criteria for inclusion in a multivariable model: body system (categorized as joints, skin and hooves, respiration, skeletal and other; p = 0.057), if a diagnostic code was written out in the VCR (p < 0.001) and whether it was a first or a follow up visit (p < 0.001). The two-way interaction between presence/absence of a diagnostic code in the VCR and type of visit was non-significant and thus not included in the model. Including clinic as a random factor was non-significant (accounted for 1% of the model variance) and was also excluded from the model. The results from the logistic model are presented in table 4. Factors associated with a high correctness on diagnostic information included a diagnostic code written in the VCR and being a first visit. The value of the Hosmer-Lemeshow goodness-of-fit statistic was 1.91 and the corresponding p-value computed from the chi-square distribution with 6 degrees of freedom was 0.93 (i.e non-significant), indicating good model fit. The predictive ability of the model was 0.92 suggesting excellent predictive ability. The residual plots indicated that two covariate patterns (including 4 and 29 observations, respectively) were divergent. The model was rerun without the 33 divergent observations, producing a model with two of the three explanatory factors (whether a diagnostic code was written in the VCR and system diagnosis) remaining statistically significant and with same direction of the odds ratio point estimates (the model retained similar level of the Hosmer-Lemeshow goodness-of-fit test; p = 0.93, 6 df). However, there was no indication that the diverging observations were erroneous so they remained in the final model. In conclusion, the model fit was not optimal, but it was considered adequate for examining the main effects.

**Table 4:** Logistic regression analysis

Explanatory variable	Odds ratio	95% CI
Baseline		
Type of visit: First visit	3.8	1.4, 10
Follow up visit	baseline	
Explicit diagnostic code present in the VCR: yes	32	7.2, 146
No code present	baseline	
System Skin and Hooves	0.6	0.1, 2.9
System Respiration	1.0	0.2, 4.6
System Skeletal	1.5	0.3, 8.1
System Other	1.8	0.2, 17
System Whole body	13	2.7, 63
System Joints	baseline	

Modelling the effect of different fixed effects on the outcome correctness^1^, results from the multivariable logistic regression model based on one observation per visit (n = 396) in a sample of visits at 18 horse clinics in Sweden during 2002.

^1 ^Defined as the proportion of recorded disease events in the database that truly happened according to the veterinary clinical records (which were considered as the gold standard)

## Discussion

The database evaluated in the present study is a potentially valuable clinical research tool as it includes data from a network of similarly managed and staffed equine clinics distributed throughout Sweden and the clinics attend to all types of horses with various problems. Another advantage with this clinical database is that the diagnostic information is recorded using an established coding system. Lack of a standard diagnostic classification system has been proposed as a limitation of using veterinary medical records for epidemiologic research [29]. In contrast to previously published information on morbidity in insured horses in Sweden [5], the clinical database accessed in this study offers the possibility to look at all health events, including minor/low cost events, prophylactic actions such as vaccinations and all co-morbidities seen at a visit. However, factors influencing the probability for a horse to be seen at an equine clinic (e.g. severity of disease, financial or time issues) were not addressed in this study. Taken together, the two sources of insurance and clinical data can provide an in-depth insight into the diseases and health care for horses in most of Sweden. However, a formal validation of this clinical database was considered essential to identify the quality of the diagnostic information in this database in order to assess its potential use for research.

Overall, the distribution of diagnoses in the sample was reflective of that seen for all visits, indicating that the sample was representative of the medical database. For practical reasons and to minimize variability in the subjective assessment all records were evaluated solely by the principal investigator. It should be noted that the accuracy of recording data, by the veterinarian, into the VCR was not evaluated and this may be a source of error that has not been determined in this study. The estimation of number of problems in the VCR should be interpreted with care as it was influenced by the completeness of the VCR (i.e. the record writing of the veterinarian) and the interpretation of the primary investigator. In this study a fairly generous interpretation was chosen with all problems noted in the VCR counted as diagnoses/problems. In fact, more diagnoses were recorded in the VCR compared to the CCR for 12% of the visits. On several occasions this was due to the procedure teeth floating being recorded without a related diagnosis (such as pointed enamel ridges). A likely explanation to this is that teeth floating was previously performed routinely at the clinics at the horse owner's request (and less likely to be noted as a diagnosis) whereas today the company's policy is to perform teeth floating when necessary and then note a diagnosis related to the problem (personal communication, Jenny Ennerdal, ATG Equine Clinics Ltd). A similar level of under-reporting of co-morbidities has also been reported in human medical computerized record systems [30].

If the CCR was used to find cases of specific conditions (such as for retrospective studies), the overall correctness suggests that, of the 'n' visits identified, on average 92% of them would have experienced the condition of interest. The estimates of correctness of 87%, 88%, 93% and 96% for body system skin and hooves, joints, respiration and skeleton, respectively, indicate satisfactory usefulness of the database in finding cases within those systems. However, as cases of disease may be recorded as "without diagnosis" in the CCR despite specific information in the VCR (seen for two of the 17 cases of "without diagnosis" in the CCR in the assessment of correctness), there is likely an under-estimation of disease in organ systems other than whole body. The correctness was similar to the corresponding estimates presented in a validation of diagnostic information in a canine insurance database study (i.e. 90% when combining the categories agreement and minor disagreement for both types of claims versus missing data in practice records and major disagreement) [20] and that seen when disease information in human electronic patient records in England were evaluated against standard criteria for each selected diagnosis (87%) [13]. A similar level of the estimated correctness for diagnostic information was found for investigated body systems in a validation of an equine insurance data base, whereas the correctness for specific diagnoses was lower (84%) [22]. Other evaluations of computerized medical records have shown variation in agreement (i.e. correctness) for diagnostic information. For example, the proportion of agreement varied between morbidities[11,22]. Further, the quality of the recording was higher for conditions with more specific diagnostic criteria, such as asthma [31].

The generally high level of completeness (91%) of the CCR indicates that overall only 9% of true cases according to VCR would not be captured by the CCR. However, for the four selected body systems, the completeness varied greatly (e.g. only 71% of respiration problems in the VCR were captured by the CCR) as the selected body systems only accounted for some of the diagnoses. This indicates that the ability to find desired cases will depend upon affected system and likely also on the specific disease problem involved. In general, the major concern related to whether the cases found were typical of all cases, or whether there was some bias that resulted in the errors. Based on the assessment it appeared that there were less severe and non-clinically important problems that were not recorded in the CCR (data not shown). In fact, for system respiration, the cases in the VCR that were not recorded in the CCR as respiration problems were generally minor and often co-morbidities. Further, some respiration cases (according to the classification criteria used in the study and based on the information in the VCR) were designated "without diagnosis" (in system whole body) in the CCR when the VCR information indicated respiration (data not shown). This error may be related to the use of the diagnostic registry but also to the veterinarian's subjective impression of the case, which may be difficult to assess based solely on the VCR information and the classification criteria. The completeness (sensitivity) for the evaluated systems was generally higher and correctness (positive predictive value) overall similar to estimates presented for insurance data [22]. Combining findings on the correctness and completeness it is suggested that problems in body system joints will be accurately captured by searches in the CCR. However, for other body system, the ability of CCR to capture true cases (according to the VCR) seems less satisfactory, especially with respect to the level of completeness. Overall, it is likely that disease events with more severe/acute signs will be recorded in the CCR, as they may have been the main reason for a clinic visit. In addition such events may have been more thoroughly investigated and hence provide a more specific diagnosis in the relevant body system. Moreover, misclassification may prevent finding cases in the CCR, as has been found elsewhere when a medical database was searched to identify cases and controls in a study on cervical vertebral compressive myelopathy in horses [14]. Varying levels of completeness has been reported. For example, a completeness of 71% was seen when disease information in a dairy disease database was compared to farmers' disease records in Sweden [19]. Further, similar and higher levels of completeness was reported from investigation of computer recording of two specific diseases in four human general practices (92 and 97%, respectively)[32].

For the systems evaluated, the prevalence in the two sources agreed rather well. This was expected as the two sources are linked since the computerized information is based on the VCR information (i.e. the gold standard), with the possibility to record all problems present in the VCR. The evaluation of the four body systems investigated was somewhat limited by the few number of observations within each system. Distinction between skin and hooves would have facilitated comparison with the validation of equine medical insurance data [22] but this was barred by the low number of observations in this system in the present study. However, similar ranking of the most commonly registered systems was seen when the correctness of computerized equine medical insurance data was evaluated [22]. The exceptions were the digestive system which was the second most common system in insurance data (in 61 of 540 claims) but ranked lower in this database, and the respiratory system which was not among the most commonly recorded systems in insurance data. This difference may be explained by that some cases associated with high cost and need for intensive care (e.g. many cases of colic and diarrhoea) are in general not treated at the ATG Equine Clinics (i.e. they were day-clinics without provision of longer term hospital care) whereas such cases will almost invariably be captured by insurance data on insured horses. On the contrary, the lower frequency of respiratory problems in the insurance database could reflect that many respiratory problems are low-cost events or that different types of horses are included in the respective study populations. On the contrary, other problems that have been noted as common by horse owners in other parts of the world, such as skin disease (proportional morbidity = 14%) and eye disorders (proportional morbidity = 10%) [33] will more likely be captured by clinical data. Thus, combining information in clinical and insurance data will increase the possibility to adequately describe the health situation of Swedish horses.

To improve the usefulness of the database it is important that the most accurate (i.e. complete and correct) diagnostic information is recorded at each visit. Factors related to correct and complete recording of diagnostic information in the CCR include the classification system (i.e. the diagnostic registry) and individual interest by the veterinarian to correctly classify the problem, correctly interpret and transfer the information into the database; all subjective and personal criteria. It is therefore of key importance to engage the veterinarians and other clinic staff in clinical record writing so that complete and specific (to the extent this is possible) information on the diagnostic information is recorded at every visit. Using the data and informing the clinicians of the potential usefulness of the information relevant to their daily work might motivate them to improve recording. Further, this medical information is of vital importance in conducting evidence based medicine, so that the astute clinician can accurately follow disease treatment methods and outcomes of all relevant clinic patients. It is recommended by the company that the veterinarian records the exact code in the VCR but the extent to which this is followed varies. As well, there are weaknesses in the data recording (the CCR) due to variation in amount of initiative for record transfer by the attending veterinarian and/or technical assistants. The use of an "unspecific diagnosis" by veterinarians has also been illustrated in a study where a higher proportion of cases with the unspecific diagnosis "other disorders" was observed in a dairy industry cattle database (based on veterinary reporting) than in farmers' records [19]. Lack of transfer and miscoding of information has been reported as the most frequent reasons for discrepancies in computerized medical records at a veterinary teaching hospital [18].

The evaluation of the information on affected limb (including only cases with correctness on diagnosis) showed overall good correctness (>95%). The four cases of discrepancy between the sources were due to the affected limb information in the CCR not being updated at a follow up visit but re-recording of the affected limb information noted at an earlier visit.

In general, the results of the logistic model were supported by the univariable analyses. For the logistic model, the lack of model improvement when including clinic as a random factor is a positive finding for the company ATG as is indicates that the individual clinics do not differ significantly regarding the quality of the registered disease information. However, this finding may also relate to the design of the study (e.g. sample size, classification criteria). To account for clustering within visit was not possible due to the large proportion of visits having only one diagnosis recorded (82%) and that the number of observations within each cluster was small (<5). Although not optimal, it was therefore decided to perform the logistic model on a subset of data including one observation per visit. Thus using single-level logistic regression would likely not bias the standard errors downwards (Type I error) [34].

Large clinical databases offer access to an amount of data not possible to retrieve elsewhere and may be an important source in defining health issues in the horse population. However, formal validation of any database is a necessary step in investigating its usefulness for research purposes and to identify its strengths and limitations.

## Conclusion

Overall, the completeness and correctness of the diagnostic information in this clinical database was excellent, although varying according to body system affected. Based on the overall high level of correctness and completeness the database was considered useful for research purposes, with the caveat that diseases of the respiratory system appeared to lack completeness of transfer of diagnostic information data to the CCR. For the body systems investigated, the highest level of completeness was seen for joints whereas correctness was highest for respiration. In general, it is suggested that the veterinarian sets a more informative diagnosis whenever possible instead of recording "without diagnosis". Accurate recording of disease information will improve the usefulness of the database, facilitate clinical management and promote utility of data for research purposes. Finally, it appears that the ATG equine CCR database reflects the VCR data well and the information was considered useful for research purposes with due considerations for the types of problems treated at these clinics.

## Competing interests

This study has been supported financially by ATG.

## Authors' contributions

JCP planned and carried out the study, managed the material, performed all the statistical analyses and prepared the manuscript. AE, JP and BB participated in the design and carrying out of the study, and in interpretation of the results and drafting the manuscript. All authors read and approved the final manuscript.

## References

[B1] Lawrenson R Williams T Farmer R Clinical information for research; the use of general practice databases J Public Health Med 1999 21 299 304 10.1093/pubmed/21.3.299 10528957

[B2] Miller DR Wang F West W Safford M Pogach L Using computerized medical records to estimate disease prevalence in a dynamic population: Diabetes in the VA Am J Epidemiol 2002 155 s75 s75 10.1093/aje/155.6.534

[B3] Hamilton W Lancashire R Sharp D Peters T Cheng K Marshall T The risk of colorectal cancer with symptoms at different ages and between the sexes: a case-control study BMC Med 2009 7 17 10.1186/1741-7015-7-17 19374736 PMC2675533

[B4] Culp WT Zeldis TE Reese MS Drobatz KJ Primary bacterial peritonitis in dogs and cats: 24 cases (1990-2006) J Am Vet Med Assoc 2009 234 906 913 10.2460/javma.234.7.906 19335241

[B5] Egenvall A Penell JC Bonnett BN Olson P Pringle J Morbidity of Swedish horses insured for veterinary care between 1997 and 2000: variations with age, sex, breed and location Vet Rec 2005 157 436 443 16215244 10.1136/vr.157.15.436

[B6] Hennessy S Bilker WB Weber A Strom BL Descriptive analyses of the integrity of a US Medicaid claims database Pharmacoepidemiol Drug Saf 2003 12 103 111 10.1002/pds.765 12647699

[B7] Lewis JD Brensinger C Bilker WB Strom BL Validity and completeness of the General Practice Research Database for studies of inflammatory bowel disease Pharmacoepidemiol Drug Saf 11 211 218 10.1002/pds.698 12051120

[B8] Lawrenson R Todd JC Leydon GM Williams TJ Farmer RDT Validation of the diagnosis of venous thromboembolism in general practice database studies Br J Clin Pharmacol 2000 49 591 596 10.1046/j.1365-2125.2000.00199.x 10848723 PMC2015047

[B9] Ray WA Griffin MR Fought RL Adams ML Identification of Fractures from Computerized Medicare Files J Clin Epidemiol 1992 45 703 714 10.1016/0895-4356(92)90047-Q 1619449

[B10] Fombonne E Heavey L Smeeth L Rodrigues LC Cook C Smith PG Meng L Hall AJ Validation of the diagnosis of autism in general practitioner records BMC Public Health 2004 4 5 10.1186/1471-2458-4-5 15113435 PMC394332

[B11] Roos LL Sharp SM Cohen MM Comparing Clinical Information with Claims Data - Some Similarities and Differences J Clin Epidemiol 1991 44 881 888 10.1016/0895-4356(91)90050-J 1890430

[B12] Pilpel D Fraser GM Kosecoff J Brook RH Validation of A Centrally Maintained Computerized Hospital Database - Comparison with Operating-Room Logbooks Isr J Med Sci 1993 29 287 291 8314689

[B13] Hassey A Gerrett D Wilson A A survey of validity and utility of electronic patient records in a general practice Br Med J 2001 322 1401 1405 10.1136/bmj.322.7299.1401 PMC32256 11397747

[B14] Levine JM Ngheim PP Levine GJ Cohen ND Associations of sex, breed, and age with cervical vertebral compressive myelopathy in horses: 811 cases (1974-2007) J Am Vet Med Assoc 2008 233 1453 1458 10.2460/javma.233.9.1453 18980501

[B15] Couetil LL Ward MP Analysis of risk factors for recurrent airway obstruction in North American horses: 1,444 cases (1990-1999) J Am Vet Med Assoc 2003 223 1645 1650 10.2460/javma.2003.223.1645 14664454

[B16] Dugdale AH Langford J Senior JM Proudman CJ The effect of inotropic and/or vasopressor support on postoperative survival following equine colic surgery Vet Anaesth Analg 2007 34 82 88 10.1111/j.1467-2995.2006.00299.x 17316388

[B17] Mohammed HO Rebhun WC Antczak DF Factors associated with the risk of developing sarcoid tumours in horses Equine Vet J 1992 24 165 168 1606927 10.1111/j.2042-3306.1992.tb02808.x

[B18] Pollari FL Bonnett BN Allen DG Bamsey SC Martin SW Quality of computerized medical record abstract data at a veterinary teaching hospital Prev Vet Med 1996 27 141 154 10.1016/0167-5877(95)01004-1

[B19] Mörk M Lindberg A Alenius S Vagsholm I Egenvall A Comparison between dairy cow disease incidence in data registered by farmers and in data from a disease-recording system based on veterinary reporting Prev Vet Med 2009 88 298 307 10.1016/j.prevetmed.2008.12.005 19178966 PMC7114122

[B20] Egenvall A Bonnett BN Olson P Hedhammar A Validation of computerized Swedish dog and cat insurance data against veterinary practice records Prev Vet Med 1998 36 51 65 10.1016/S0167-5877(98)00073-7 9677627

[B21] Nodtvedt A Bergvall K Emanuelson U Egenvall A Canine atopic dermatitis: validation of recorded diagnosis against practice records in 335 insured Swedish dogs Acta Vet Scand 2006 48 8 10.1186/1751-0147-48-8 16987404 PMC1553461

[B22] Penell JC Egenvall A Bonnett BN Pringle J Validation of computerized Swedish horse insurance data against veterinary clinical records Prev Vet Med 2007 82 236 251 10.1016/j.prevetmed.2007.05.020 17644201

[B23] Swedish Animal Hospital Association O Diagnostic registry for the horse, the dog and the cat (Diagnosregister för häst, hund och katt) Tåberg, Tåbergs tryckeri, Sweden 1993 1

[B24] Penell JC Egenvall A Bonnett BN Olson P Pringle J Specific causes of morbidity among Swedish horses insured for veterinary care between 1997 and 2000 Vet Rec 2005 157 470 477 16227382 10.1136/vr.157.16.470

[B25] Dohoo I Martin W Stryhn H Screening and diagnostic tests Veterinary Epidemiologic Research 2003 Charlottetown, Prince Edward Island, Canada: AVC inc 85 120

[B26] Dohoo I MW Stryhn H Mixed Models for Discrete Data Veterinary Epidemiologic Research 2003 Charlottetown, Prince Edward Island, Canada: AVC inc 499 520

[B27] Snijders T Bosker R Multilevel Analysis: An Introduction to Basic and Advanced Multilevel Modeling 1999 London: SAGE

[B28] Hosmer DW LS Assessing the Fit of the Model Applied Logistic Regression 2000 Second New York: John Wiley & Sons, Inc 143 202 full_text

[B29] Brouwer H Schouten EG Noordhuizen JPTM Vanvoorthuysen PF Potential of Computerized Medical Records for Epidemiologic Research Tijdschr Diergeneeskd 1995 120 296 2997761967

[B30] Lloyd SS Rissing JP Physician and coding errors in patient records JAMA 254 1330 1336 10.1001/jama.254.10.1330 3927014

[B31] Jordan K Porcheret M Croft P Quality of morbidity coding in general practice computerized medical records: a systematic review Fam Pract 2004 21 396 412 10.1093/fampra/cmh409 15249528

[B32] Pringle M Ward P Chilvers C Assessment of the completeness and accuracy of computer medical records in four practices committed to recording data on computer Br J Gen Pract 1995 45 537 541 7492423 PMC1239405

[B33] Cole FL Hodgson DR Reid SW Mellor DJ Owner-reported equine health disorders: results of an Australia-wide postal survey Aust Vet J 2005 83 490 495 10.1111/j.1751-0813.2005.tb13301.x 16119422

[B34] Clarke P When can group level clustering be ignored? Multilevel models versus single-level models with sparse data J Epidemiol Community Health 2008 62 752 758 10.1136/jech.2007.060798 18621963

